# A PARALIND Decomposition-Based Coherent Two-Dimensional Direction of Arrival Estimation Algorithm for Acoustic Vector-Sensor Arrays

**DOI:** 10.3390/s130405302

**Published:** 2013-04-19

**Authors:** Xiaofei Zhang, Min Zhou, Jianfeng Li

**Affiliations:** 1 College of Electronic and Information Engineering, Nanjing University of Aeronautics & Astronautics, Nanjing 210016, China; E-Mails: fei_zxf@163.com (X.Z.); zm_nuaa2012@163.com (M.Z.); 2 Nanjing Panda Electronics Group, Nanjing 210008, China; 3 Laboratory of Modern Acoustic of Ministry of Education, Nanjing University, Nanjing 210093, China

**Keywords:** arbitrary array, acoustic vector-sensor, coherent angle estimation, PARALIND decomposition

## Abstract

In this paper, we combine the acoustic vector-sensor array parameter estimation problem with the parallel profiles with linear dependencies (PARALIND) model, which was originally applied to biology and chemistry. Exploiting the PARALIND decomposition approach, we propose a blind coherent two-dimensional direction of arrival (2D-DOA) estimation algorithm for arbitrarily spaced acoustic vector-sensor arrays subject to unknown locations. The proposed algorithm works well to achieve automatically paired azimuth and elevation angles for coherent and incoherent angle estimation of acoustic vector-sensor arrays, as well as the paired correlated matrix of the sources. Our algorithm, in contrast with conventional coherent angle estimation algorithms such as the forward backward spatial smoothing (FBSS) estimation of signal parameters via rotational invariance technique (ESPRIT) algorithm, not only has much better angle estimation performance, even for closely-spaced sources, but is also available for arbitrary arrays. Simulation results verify the effectiveness of our algorithm.

## 1. Introduction

Compared with traditional acoustic pressure sensor arrays, acoustic vector sensors can measure the acoustic pressure as well as all three orthogonal components of the acoustic particle velocity at a single point in space, which offers certain significant advantages in collecting more acoustic information and enhancing the system performance [1–6]. Since the measurement model of an acoustic vector sensor array was developed in [2], researchers mainly turned to the study of incoming signal direction of arrival (DOA) estimation and have proposed many DOA estimation algorithms, which include the Capon technique [4], estimation of signal parameters via rotational invariance technique (ESPRIT) [7–9], root multiple signal classification (MUSIC) [10], self-initiating MUSIC [11], hypercomplex [12], propagator method (PM) [13], trilinear decomposition or parallel factor (PARAFAC) [14], as well as others [15–21]. The subspace-based methods, such as ESPRIT [7–9] and MUSIC [10,11], require eigenvalue decomposition (EVD) of the cross correlation matrix and singular value decomposition (SVD) of the received data to obtain the signal subspace or noise subspace, which implies fairly high computational complexity, while the propagator method (PM) is considerably less demanding because the PM does not require any EVD of the cross correlation matrix and SVD of the received data [22]. However, only in high-snapshots situation and signal to noise ratio (SNR), can the PM algorithm provide a better estimation performance. In most current algorithms for DOA estimation, some precise *a priori* knowledge, including the sensor locations, gain, phase response and mutual coupling of the receiver array is needed, but in realistic situations this can seldom be pre-known. For example, Capon [4] and the MUSIC algorithm can be used for arbitrary arrays, however they need to pre-know the array geometry. Besides, peak searching is also required for Capon and MUSIC algorithms, which renders a heavier computational cost. EVD of the cross spectral matrix or SVD of the received data to obtain the signal subspace is needed for the ESPRIT algorithm [7], which has been used for two dimensional (2D) DOA estimation for arbitrarily spaced arrays at unknown locations based on the acoustic vector-sensor property. However, a problem existing in [7] is that the ESPRIT algorithm needs an extra pair matching which increases the computational load, and usually fails to work in lower SNR when it is used for 2D-DOA estimation. Reference [14] proposes a trilinear decomposition-based 2D-DOA estimation algorithm for acoustic vector sensor arrays, which provides DOA estimation for the arbitrarily spaced sensor arrays and doesn't require knowledge of sensor locations and extra pair matching.

The reflected signal of the same sources through different propagation paths will produce multipath signals. Therefore, it is of significant importance to study the coherent angle estimation problem. The angle estimation algorithms listed above are all proposed for incoherent sources. When it comes to coherent sources, the coherency of sources will result in serious degradation or invalidity of the above algorithms. Some conventional coherent angle estimation algorithms, including forward backward spatial smoothing (FBSS) [23,24], only work for uniform arrays.

This paper combines the acoustic vector-sensor array parameter estimation problem with the so-called PARAllel profiles with LINear Dependencies (PARALIND) model, and proposes a blind coherent 2D-DOA estimation algorithm for arbitrarily spaced acoustic vector-sensor arrays subject to unknown locations by exploiting the PARALIND decomposition approach. Our algorithm can provide coherent and incoherent two-dimensional angle estimation for arbitrary arrays, and it automatically archives paired azimuth and elevation angles, and the paired correlated matrix of the sources can also be acquired. Compared with conventional coherent angle estimation algorithms such as the FBSS-ESPRIT algorithm which only works for uniform arrays, our algorithm has much better angle estimation performance. Furthermore, our algorithm performs considerably well for angle estimation of closely spaced sources. We also derive the Cramér-Rao bound (CRB) of angle estimation for arbitrarily spaced acoustic vector-sensor arrays. Simulation results verify the effectiveness of the proposed algorithm.

The trilinear decomposition, also known as PARAFAC analysis [25,26], has been naturally related to angle estimation for arbitrarily spaced acoustic vector-sensor arrays at unknown locations [14]. However, the PARAFAC angle estimation solution is usually non-unique when the coherent sources exist. The PARALIND model [27,28] is a generalization of PARAFAC suitable for solving problems with linear dependent factors where PARAFAC analysis will fail to provide meaningful results. Our work links the coherent angle estimation problem to the PARALIND model, and proposes a PARALIND decomposition-based coherent angle estimation algorithm for arbitrary arrays, which can be regarded as an extension of the work presented in [14]. Reference [27] proposed the PARALIND model for application in biology and chemistry; the present paper expands this model to the acoustic vector-sensor array signal processing problem to estimate coherent DOA and automatically achieve paired two-dimensional angle estimations, which is an innovation.

Although the ESPRIT algorithm [7] and our algorithm can be used for DOA estimation for arbitrarily spaced acoustic vector-sensor arrays, there are some differences between our paper and Reference [7]. The latter proposed an ESPRIT algorithm for DOA estimation for arbitrarily spaced three-dimensional arrays of vector hydrophones, but it fails to work well for coherent sources. Our work exploits the PARALIND decomposition approach to estimate 2D-DOA in arbitrarily spaced acoustic vector-sensor arrays with unknown locations, and our algorithm is suitable for coherent sources.

The present paper is structured as follows: Section 2 develops the data model for arbitrarily spaced acoustic vector-sensor arrays at unknown locations; Section 3 establishes our PARALIND decomposition-based coherent 2D-DOA angle estimation algorithm in addition with the identifiability issues and complexity analysis; In Section 4, simulation results are presented to verify effectiveness of the proposed algorithm, while the final conclusions are made in Section 5.

## 2. Data Model

**Figure 1.**
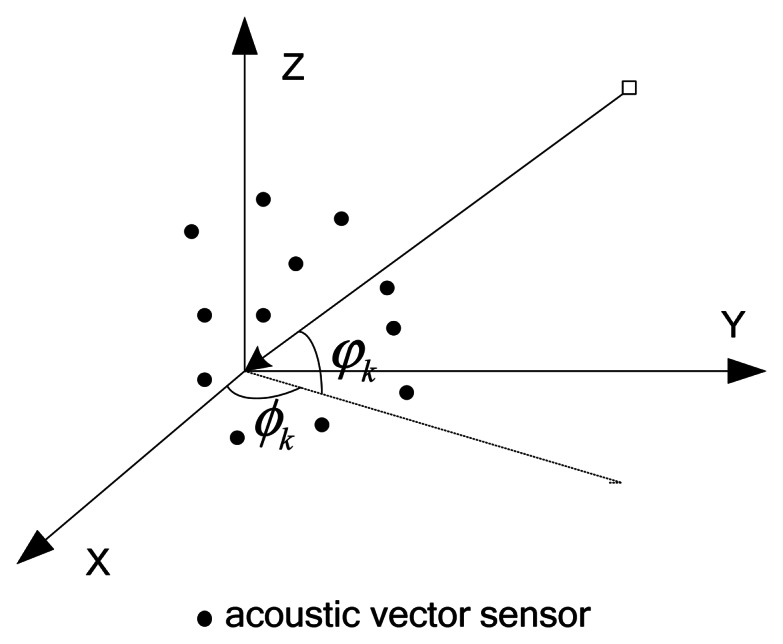
The structure of acoustic-vector sensor array [14].

We assume that a total of *K* narrowband plane waves impinge on an array equipped with *M* acoustic vector sensors, which are all located at arbitrary unknown three dimensional positions as shown in Figure 1. We define the location of the *m*th vector sensor as *r_m_* = (*x_m_*, *y_m_*, *z_m_*).

We also assume the signals in the far-field. *K* sources, including *K*_1_ incoherent sources and *K*-*K*_1_ coherent sources, are considered. Assume that the noise is additive white Gaussian, which is independent of the sources. The *k*th signal arrives from direction (*ϕ_k_*, *φ_k_*), where *ϕ_k_* and *φ_k_* respectively stand for the azimuth angle and the elevation angle. Let **θ**_k_ = [*ϕ_k_*, *φ_k_*]*^T^* as the 2D-DOA of the *k*th source. According to [2], the output at the acoustic vector sensors array can be expressed as:

(1) x(t)=(H∘A)Γs(t)+n(t)

where **A** = [**a**(**θ**_1_), **a**(**θ**_2_),…, **a**(**θ***_K_*)] is an *M* × *K* matrix composed of *K* receive steering vectors. **s**(*t*) = [s_1_(*t*), s_2_(*t*),…, s*_K_*(*t*)]*^T^* is a column vector consisting of amplitudes and phases of the *K*_1_ incoherent sources. **Γ** is the correlated matrix with *K* × *K*. **n**(*t*) is the received additive white Gaussian noise vector. **H** = [**h**_1_, **h**_2_,…, **h***_K_*] in which:

(2) $$h_{k}=\left[\begin{array}{c} 1\\ \cos\phi_{k}\cos\varphi_{k}\\ \sin\phi_{k}\cos\varphi_{k}\\ \sin\varphi_{k} \end{array}\right]$$

Therefore, the output of *J* snapshots can be given by:

(3) $$X=[x(t_{1}),x(t_{2}),\cdots ,x(t_{J})]$$

**X** can be compactly expressed as:

(4) X=(H∘A)ΓS+N

where **S** = [**s**(*t*_1_), **s**(*t*_2_),…, **s**(*t*_J_)], **N** = [**n**(*t*_1_), **n**(*t*_2_),…, **n**(*t*_J_)] is an 4*M* × *J* matrix composed of *J* snapshots of received additive white Gaussian noise.

## 3. The Coherent Two-Dimensional Angle Estimation

### 3.1. PARALIND Decomposition

Define **Ỹ** = **X***^T^*, according to the signal model in Equation (4), **Ỹ** can be expressed as:

(5) $$\tilde{Y}={\left(\Gamma S\right)}^{T}{\left(H\circ A\right)}^{T}+N^{T}$$

According to [27], least fitting for the signal model in Equation (5) amounts to:

(6) $$\min_{\Gamma ,S,H,A}{\left\|\tilde{Y}-{\left(\Gamma S\right)}^{T}{\left(H\circ A\right)}^{T}\right\|}_{F}$$

In a no-noise case, according to Equation (5), we have:

(7) $$\begin{array}{ll} Y & =[Y_{1},Y_{2},Y_{3},Y_{4}]\\ & =[{\left(\Gamma S\right)}^{T}D_{1}(H)A^{T},{\left(\Gamma S\right)}^{T}D_{2}(H)A^{T},{\left(\Gamma S\right)}^{T}D_{3}(H)A^{T},{\left(\Gamma S\right)}^{T}D_{4}(H)A^{T}] \end{array}$$

where **Y***_n_* = (**ΓS**)*^T^D_n_*(**H**)**A***^T^* ∈ **C**^*J*×*M*^, *n* = 1,2,3,4.. Then we have:

(8) $$\text{vec}(Y_{n})=\text{vec}({\left(\Gamma S\right)}^{T}D_{n}(H)A^{T})=\left(AD_{n}(H)⊗S^{T}\right)\text{vec}(\Gamma^{T})$$

According to [7], stacking these vectors leads to:

(9) $$\left[\begin{array}{l} \text{vec}(Y_{1})\\ \text{vec}(Y_{2})\\ \text{vec}(Y_{3})\\ \text{vec}(Y_{4}) \end{array}\right]=\left[\begin{array}{l} AD_{1}(H)⊗S^{T}\\ AD_{2}(H)⊗S^{T}\\ AD_{3}(H)⊗S^{T}\\ AD_{4}(H)⊗S^{T} \end{array}\right]\;\text{vec}(\Gamma^{T})$$

Equation (9) can be expressed compactly as:

(10) $$\text{vec}(Y)=\left[(H\circ A)⊗S^{T}\right]\;\text{vec}(\Gamma^{T})$$

According to Equation (10), we can obtain:

(11) $$\text{vec}(\Gamma^{T})={\left[(H\circ A)⊗S^{T}\right]}^{+}\text{vec}(Y)$$

For attaining *vec*(**Γ***^T^***)**, then **Γ** can be easily updated via transforming the column vector to its original column-wise matrix.

According to Equation (6), the least square (LS) update for **S***^T^* is given by:

(12) $$S^{T}=Y{\left({\left((H\circ A)\Gamma \right)}^{T}\right)}^{+}$$

According to Equation (7), we have:

(13) $$\sum_{n=1}^{4}{Y_{n}}^{H}Y_{n}=\left(\sum_{n=1}^{4}{Y_{n}}^{H}{\left(\Gamma S\right)}^{T}D_{n}(H)\right)A^{T}=A^{*}\;(\sum_{n=1}^{4}{D_{n}}^{*}(H){\left(\Gamma S\right)}^{*}{\left(\Gamma S\right)}^{T}D_{n}(H))A^{T}$$

**A** has full column rank, so we can acquire A* via:

(14) $$\begin{array}{l} \begin{array}{cc} A^{*} & =\left(\sum_{n=1}^{4}{Y_{n}}^{H}{\left(\Gamma S\right)}^{T}D_{n}(H)\right){\left(\sum_{n=1}^{4}{D_{n}}^{*}(H){\left(\Gamma S\right)}^{*}{\left(\Gamma S\right)}^{T}D_{n}(H)\right)}^{-1}\\ & =\left(\sum_{n=1}^{4}{Y_{n}}^{H}{\left(\Gamma S\right)}^{T}D_{n}(H)\right){\left(\sum_{n=1}^{4}\left({\left(\Gamma S\right)}^{*}{\left(\Gamma S\right)}^{T}\right)⊙\left(H^{H}H\right)\right)}^{-1} \end{array} \end{array}$$

Similarly, we have:

(15) $${\left(\Gamma S\right)}^{*}Y_{n}A={\left(\Gamma S\right)}^{*}{\left(\Gamma S\right)}^{T}D_{n}(H)A^{T}A$$

Extracting the diagonal elements of the matrices in two sides of the equation, we get:

(16) $$\text{dia}g^{-1}\left({\left(\Gamma S\right)}^{*}Y_{n}A\right)=\left({\left(\Gamma S\right)}^{*}{\left(\Gamma S\right)}^{T}\right)⊙\left(A^{T}A\right)\text{dia}g^{-1}\left(D_{n}(H)\right)$$

Then we get:

(17) $$\text{dia}g^{-1}\left(D_{n}(H)\right)={\left(\left({\left(\Gamma S\right)}^{*}{\left(\Gamma S\right)}^{T}\right)⊙\left(A^{T}A\right)\right)}^{-1}\text{dia}g^{-1}\left({\left(\Gamma S\right)}^{*}Y_{n}A\right)$$

The matrix **H** can be straightforwardly obtained via *diag*^−1^(*D*_n_(**H**)), *n* = 1,2,3,4.

According to Equations (11), (12), (14), and (17), we can show PARALIND algorithm applied in the data model established in this paper in detail as follows:

According to Equation (11), the update for *vec*(**Γ̂***^T^*) is given by:

(18) $$\text{vec}({\hat{\Gamma }}^{T})={\left[(\hat{H}\circ \hat{A})⊗{\hat{S}}^{T}\right]}^{+}\text{vec}(\tilde{Y})$$

where **Ỹ** is the noisy signal. **Ĥ**, **Â**, and **Ŝ***^T^* are the previously obtained estimates of **H**, **A**, and **S***^T^*, respectively. According to Equation (12), the LS update for **S***^T^* is obtained via:

(19) $${\hat{S}}^{T}=\tilde{Y}{\left({\left((\hat{H}\circ \hat{A})\hat{\Gamma }\right)}^{T}\right)}^{+}$$

where **Ĥ**, **Â**, and **Γ̂** are the previously obtained estimates of **H**, **A**, and **Γ**, respectively. According to Equation (14), the update for A* is shown as:

(20) $${\hat{A}}^{*}=\left(\sum_{n=1}^{4}{{\tilde{Y}}_{n}}^{H}{\left(\hat{\Gamma }\hat{S}\right)}^{T}D_{n}(\hat{H})\right){\left(\sum_{n=1}^{4}\left({\left(\hat{\Gamma }\hat{S}\right)}^{*}{\left(\hat{\Gamma }\hat{S}\right)}^{T}\right)⊙\left({\hat{H}}^{H}\hat{H}\right)\right)}^{-1}$$

where **Ỹ***_n_* denotes the noisy signal. **Ĥ**, **Ŝ**, and **Γ̂** are the previously obtained estimates of **H**, **S**, and **Γ**, respectively. According to Equation (17), the update for *D_n_*(H) is:

(21) $$\text{dia}g^{-1}\left(D_{n}(\hat{H})\right)={\left(\left({\left(\hat{\Gamma }\hat{S}\right)}^{*}{\left(\hat{\Gamma }\hat{S}\right)}^{T}\right)⊙\left({\hat{A}}^{T}\hat{A}\right)\right)}^{-1}\text{dia}g^{-1}\left({\left(\hat{\Gamma }\hat{S}\right)}^{*}{\tilde{Y}}_{n}\hat{A}\right)$$

where **Â**, **Ŝ**, and **Γ̂** are the previously obtained estimates of **A**, **S**, and **Γ**, respectively. Finally, the update of **H**, noted as **Ĥ**, can be straightforwardly obtained via *diag*^−1^(*D_n_*(**Ĥ**)), *n* = 1,2,3,4.

Define **E** = **Ỹ** × (**Γ̂Ŝ**)*^T^* (**Ĥ** ○ **Â**)*^T^*, where **Â**, **Ĥ**, **Ŝ**, and **Γ̂** present estimates of **A**, **H**, **Γ** and **S**, respectively. The sum of squared residuals (SSR) in PARALIND model is defined as $\text{SSR}=\sum_{j=1}^{J}\sum_{i=1}^{MN}\left|e_{ji}\right|$, where *e_ji_* is the (*j*, *i*) element of the matrix **E**. According to Equations (18), (19), (20) and (21), the matrices **A**, **H**, **Γ**, and **S** are updated until the SSR ≤ 10^−8^, finally we obtain **Â**, **Ĥ**, **Ŝ**, and **Γ̂**.

### 3.2. Uniqueness of PARALIND Decomposition

According to [27], we derive the uniqueness of PARALIND decomposition in an acoustic vector-sensor array. The signal matrix in Equation (7) can be transformed to another equivalent matrix via column and row exchanging, which can be expressed as:

(22) $$\begin{array}{ll} Z & =[Z_{1},Z_{2},\cdots ,Z_{J}]\\ & =[HD_{1}({\left(\Gamma S\right)}^{T})A^{T},HD_{2}({\left(\Gamma S\right)}^{T})A^{T},\cdots ,HD_{J}({\left(\Gamma S\right)}^{T})A^{T}] \end{array}$$

The two slices **Z***_i_* and **Z***_j_* (*i* ≠ *j*) in Equation (22) are represented as:

(23) $$Z_{i}=HD_{i}({\left(\Gamma S\right)}^{T})A^{T}=HS_{E};\;Z_{j}=HD_{j}({\left(\Gamma S\right)}^{T})A^{T}=H\Lambda S_{E}$$

where **S**_E_ = *D_i_*((**ΓS**)*^T^*)**A***^T^*, and **Λ** = *D*_j_((**ΓS**)*^T^*)*D_i_*^−^^1^((**ΓS**)*^T^*). Then we form the following matrix:

(24) $$\left[\begin{array}{l} Z_{i}\\ Z_{j} \end{array}\right]=\left[\begin{array}{l} H\\ H\Lambda \end{array}\right]S_{E}$$

**H** being full row rank assures that $\text{span}(U)=\text{span}\left(\left[\begin{array}{l} H\\ H\Lambda \end{array}\right]\right)$, where **U** consists of largest *K* left singular vectors of the matrix $\left[\begin{array}{l} Z_{i}\\ Z_{j} \end{array}\right]$. The matrix **U** can be denoted as:

(25) $$U=\left[\begin{array}{l} U_{1}\\ U_{2} \end{array}\right]=\left[\begin{array}{l} H\\ H\Lambda \end{array}\right]T$$

where **T** is a nonsingular matrix. Construct auto and cross correlation matrices as follows:

(26) $$\begin{array}{c} R_{1}={U_{1}}^{H}U_{1}=T^{H}H^{H}HT=GT\\ R_{2}={U_{1}}^{H}U_{2}=T^{H}H^{H}H\Lambda T=G\Lambda T \end{array}$$

where **G** = **T***^H^***H***^H^***H**, **R**_1_ and **R**_2_ are full rank. According to Equation (26), we obtain:

(27) $$R_{2}{R_{1}}^{-1}G=G\Lambda$$

**Λ** and **G** consist of the eigenvalues and the corresponding eigenvectors of the matrix **R**_2_**R**_1_^−1^. **Λ** is unique, and **G** is recovered with the scale ambiguity and permutation ambiguity. Then **T** = **G**^−1^**R**_1_, **H** = **U**_1_**T**^−1^, **S***_E_* = **H**^+^**Z***_i_*, **A***^T^* = *D_i_*^−^^1^((**ΓS**)*^T^*) **S***_E_*.

Notably, scale ambiguity and permutation ambiguity are inherent to the separation problem. However, the scale ambiguity can be resolved easily by normalization, while the existence of permutation ambiguity is not considered for angle estimation.

### 3.3. Two-Dimensional Angle Estimation

By imposing PARALIND decomposition for the received data matrix, we get the estimate of matrix **Ĥ** = [**ĥ**_1_,**ĥ**_2_, ⋯,**ĥ***_K_*]. According to Equation (2), **ĥ***_K_* can be expressed as:

(28) $${\hat{h}}_{k}={\left[1,\cos{\hat{\phi }}_{k}\cos{\hat{\varphi }}_{k},\sin{\hat{\phi }}_{k}\cos{\hat{\varphi }}_{k},\sin{\hat{\varphi }}_{k}\right]}^{T}$$

where **ĥ***_K_* is the normalized *k*th column vector of the estimated **Ĥ**. Then the elevation angle estimate of *k*th source can be easily obtained via:

(29) $${\hat{\varphi }}_{k}=\sin^{-1}({\hat{h}}_{k}(4))$$

Finally, the azimuth angle estimate of *k*th source can be attained via:

(30) $${\hat{\phi }}_{k}=\text{angle}\left({\hat{h}}_{k}(2)+j{\hat{h}}_{k}(3)\right)$$

Obviously, the azimuth angle and elevation angle are automatically paired. We also obtain the estimate of the correlated matrix **Γ** from the PARALIND decomposition, because **Γ** is a matrix whose elements are 0 or 1, so we can easily decide it by a decision function to obtain accurate estimated **Γ̂**.

### 3.4. The Procedures of the Proposed Algorithm

Till now, we have achieved the proposal for the proposed coherent 2D-DOA estimation algorithm for the acoustic vector-sensor array. We show major steps of the proposed algorithm as follows:

Step 1: Obtain **Ỹ** from the received data matrix **X**, and then initialize for the matrices **H**, **A**, **Γ**, and **S**.
Step 2: According to Equations (18), (19), (20) and (21), update the matrices **H**, **A**, **Γ**, and **S** until *SSR* ≤ 10^−8^, finally obtain the corresponding estimated **Ĥ**, **Â**, **Γ̂**, and **Ŝ**.
Step 3: Estimate the two-dimensional DOA through Equations (28)-(30), then obtain accurate estimate **Γ̂** by decision.

In our algorithm, the complexity of each iteration is *O*(4*MJ*(2*K*^2^*K*_1_^2^ + 2*KK*_1_ + 2*K* + *K*_1_) + 4*M*(2*K*_1_^2^ + *K*^2^ + *KK*_1_ + 2*K*) + *K*^2^(*5J* + *M* + 22) + *K*^3^*K*_1_^3^ + 2*K*^3^+ *K*_1_^3^ + 5*KK*_1_*J*). The average number of iterations required for our PARALIND decomposition algorithm is about a dozen.

**Remark A:** Our algorithm obtains the estimate of correlated matrix **Γ** from PARALIND decomposition, even for partly coherent sources, the correlated matrix **Γ** can be acquired as well.

**Remark B:** The PARALIND decomposition brings the same permutation ambiguity for the estimated **Ĥ**, **Â**, and **Γ̂**, so the elevation and azimuth angles and the correlated matrix are automatically paired.

**Remark C:** If the number of sources *K* is unknown, it can be estimated by performing SVD for a slice **Z***_i_* in Equation (22) and finding the number of largest singular values.

### 3.5. CRB and Advantages of the Proposed Algorithm

According to [29], we can derive the CRB of coherent angle estimation for the acoustic vector-sensor array with unknown locations as:

(31) CRB=σ22J{Re[(DHΠG⊥D)⊙PT]}−1

where *σ*^2^ stands for the covariance of the noise and *J* denotes the number of snapshots: $D=\left[\frac{\partial u_{1}}{\partial \phi_{1}},\cdots ,\frac{\partial u_{K}}{\partial \phi_{K}},\frac{\partial u_{1}}{\partial \varphi_{1}},\cdots ,\frac{\partial u_{K}}{\partial \varphi_{K}}\right]$ with **u***_k_* = **Q**(:,*k*) and **Q** = **H** ○ **A**, $\Pi_{G}^{⊥}=\left[\begin{array}{ccc} \Pi_{Q}^{⊥} & & 0\\ & \ddots & \\ 0 & & \Pi_{Q}^{⊥} \end{array}\right]$ with $\Pi_{Q}^{⊥}=I_{4M}-Q{\left(Q^{H}Q\right)}^{-1}Q^{H}$, $P=\left[\begin{array}{cc} P_{s} & P_{s}\\ P_{s} & P_{s} \end{array}\right]$ with $P_{s}=\sum_{j=1}^{J}b(t_{j})b{\left(t_{j}\right)}^{H}/J$ and **b**(*t_j_*) = **Γs**(*t_j_*).

The advantages of the proposed algorithm can be presented as follows, which can be verified by the simulation results in Section 4:

(1) The proposed algorithm is effective for coherent and incoherent two-dimensional angle estimation.
(2) The proposed algorithm does not require precise knowledge of the characteristics of the receiver array.
(3) The proposed algorithm can archive automatically paired angles and the corresponding correlated matrix.
(4) The proposed algorithm has much better angle estimation performance than conventional FBSS-ESPRIT algorithm which only works for uniform array.
(5) The proposed algorithm has considerably performance for angle estimation of closely spaced sources.

## 4. Simulation Results

In most of the following simulations, we assume that there are 3 sources in which only source 1 and source 3 are coherent, namely the correlated matrix is $\Gamma ={\left[\begin{array}{lll} 1 & 0 & 1\\ 0 & 1 & 0 \end{array}\right]}^{T}$.

The sources are located at angles (*ϕ*_1_,*φ*_1_) = (−15°, 10°), (*ϕ*_2_,*φ*_2_) = (25°,20°), and (*ϕ*_3_,*φ*_3_) = (35°,30°), respectively. *M*, *J*, and *K* denote the number of receive sensors, snapshots, and sources, respectively. We present 1000 Monte Carlo simulations to assess the angle estimation performance of the proposed algorithm. Define root mean squared error (RMSE) as follows:

(32) $$\text{RMSE}=\frac{1}{K}\sum_{k=1}^{K}\sqrt{\frac{1}{1000}\sum_{l=1}^{1000}{\left({\hat{\phi }}_{k,l}-\phi_{k}\right)}^{2}+{\left({\hat{\varphi }}_{k,l}-\varphi_{k}\right)}^{2}}$$

where *ϕ_k_* and *φ_k_* denote the perfect azimuth and elevation angle of *k*th source, respectively. *ϕ̂_k,l_* and *φ̂_k,l_* are the estimates of *ϕ_k_* and *φ_k_* d in the *l*th Monte Carlo trail.

**Figure 2.**
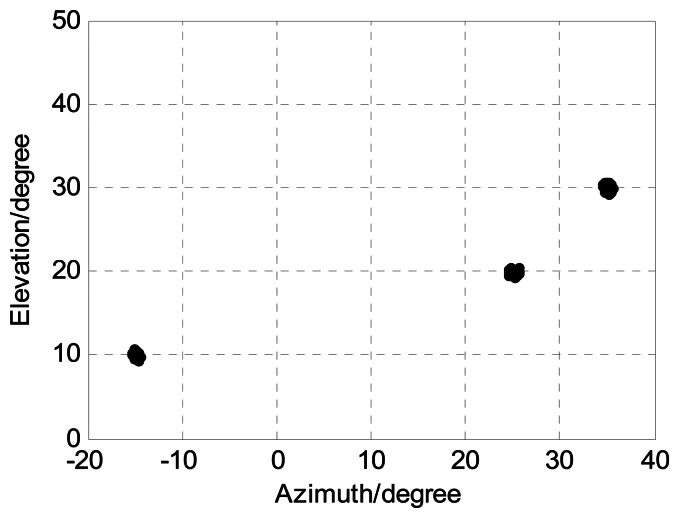
Angle estimation of our algorithm for ULA in SNR = 15 dB (*M* = 12, *J* = 100, and *K* = 3).

**Figure 3.**
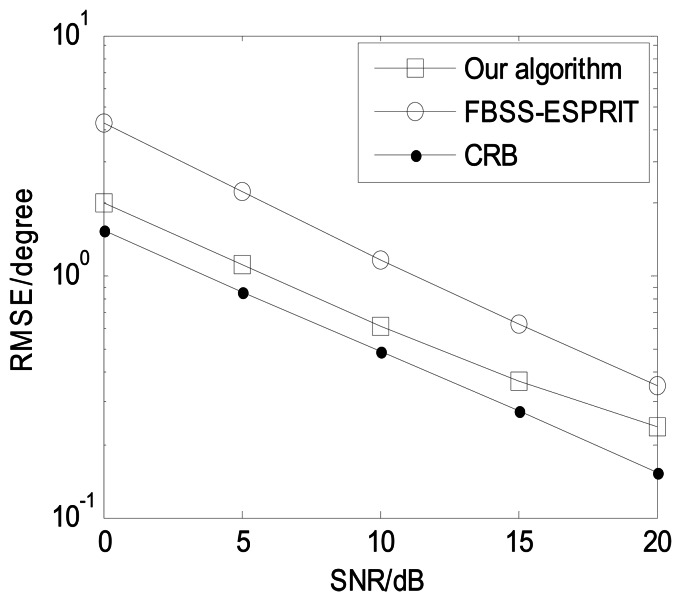
Angle estimation performance comparison for ULA (*M* = 12, *J* = 100, and *K* = 3).

In order to present the angle estimation performance comparison of the proposed algorithm and FBSS-ESPRIT algorithm, we assume that the acoustic vector-sensor array is a uniform linear array (ULA) in Figures 2 and 3. Figure 2 depicts the two-dimensional angle estimation with *M* = 12, *J* = 100, *K* = 3, and SNR = 15 dB. It illustrates that our algorithm is effective for paired two-dimensional angle estimation using ULA. Figure 3 presents the angle estimation performance comparison of the proposed algorithm, FBSS-ESPRIT algorithm, and CRB with *M* = 12, *J* = 100, and *K* = 3. It is shown that our algorithm possesses much better angle estimation performance than the FBSS-ESPRIT algorithm in the ULA situation.

**Figure 4.**
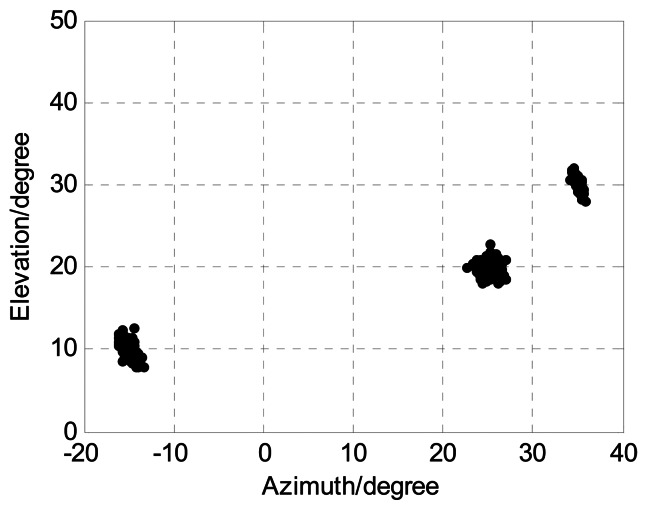
Angle estimation of our algorithm in SNR = 5 dB (*M* = 12, *J* = 100, and *K* = 3).

**Figure 5.**
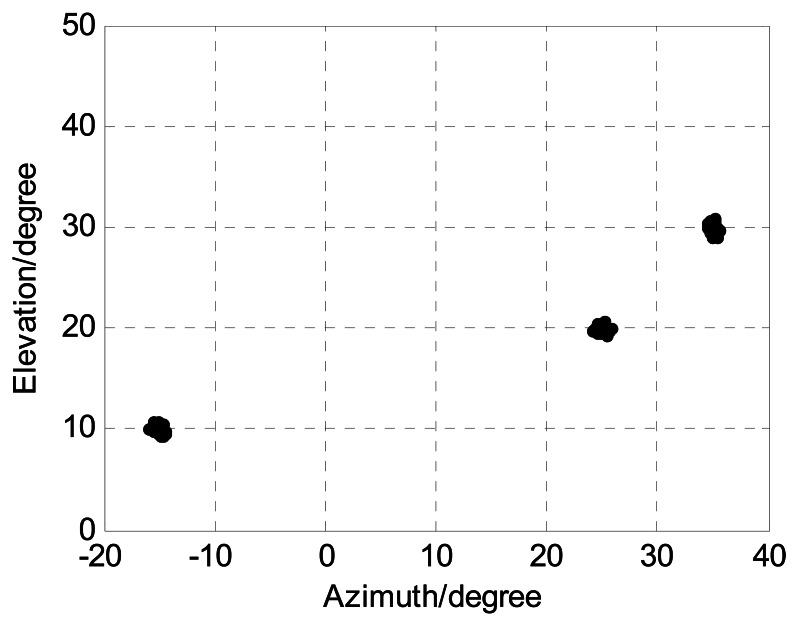
Angle estimation of our algorithm in SNR = 15 dB (*M* = 12, *J* = 100, and *K* = 3).

**Figure 6.**
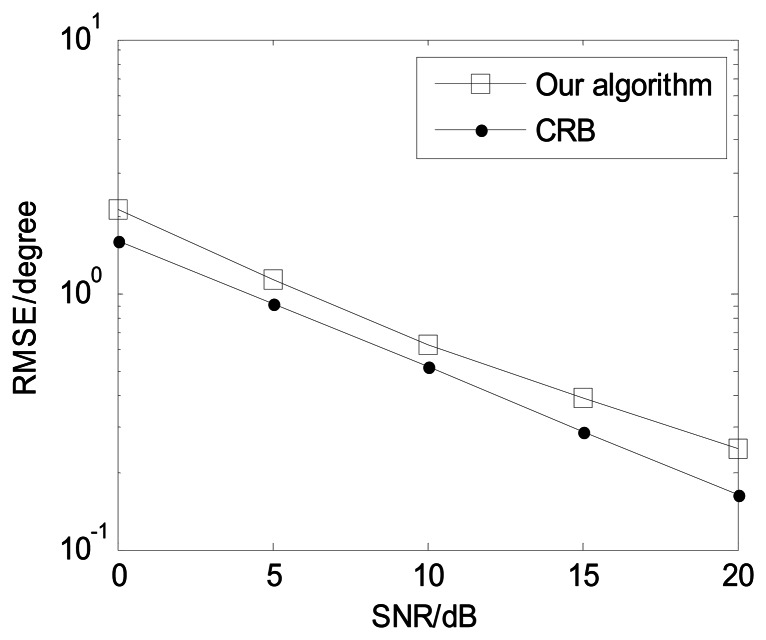
Angle estimation performance comparison (*M* = 12, *J* = 100, and *K* = 3).

**Figure 7.**
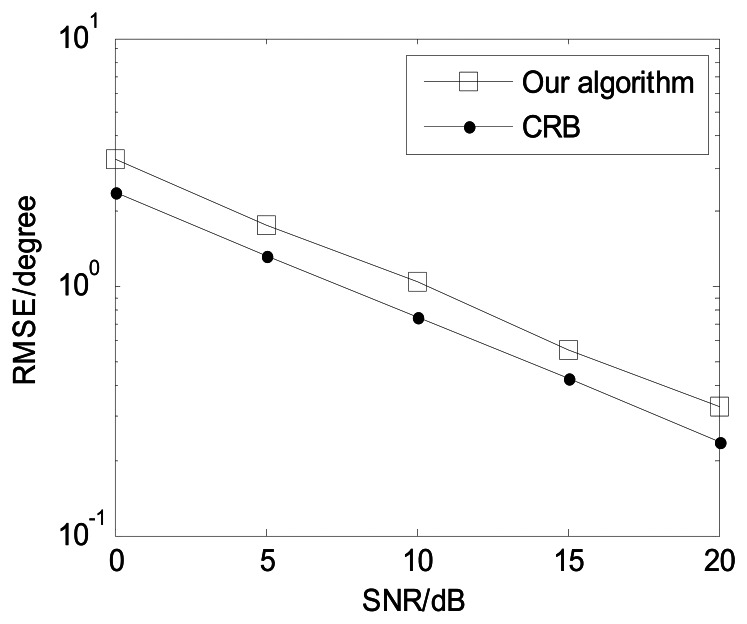
Angle estimation performance comparison (*M* = 10, *J* = 50, and *K* = 3).

**Figure 8.**
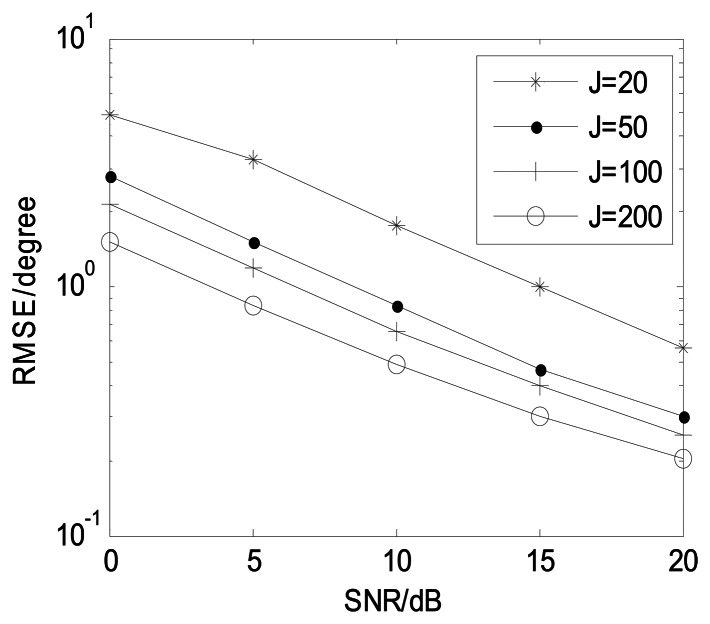
Angle estimation performance of our algorithm with different *J*. (*M* = 12, and *K* = 3).

The following Figures 4, 5, 6, 7 and 8 are for the case of arbitrarily spaced acoustic vector-sensor arrays subject to unknown locations, and we assume that the column full rank receive direction matrix is generated randomly. Figure 4 presents the two-dimensional angle estimation of the proposed algorithm with *M* = 12, *J* = 100, *K* = 3, and SNR = 5 dB, and Figure 5 depicts the angle estimation with *M* = 12, *J* = 100, *K* = 3, and SNR = 15 dB. Figures 4 and 5 illustrate that our algorithm is effective for paired two-dimensional angle estimation using arbitrary acoustic vector-sensor arrays. Figure 6 presents the angle estimation performance comparison of the proposed algorithm and CRB with *M* = 12, *J* = 100, and *K* = 3, while Figure 7 depicts the angle estimation performance comparison with *M* = 10, *J* = 50, and *K* = 3.

**Figure 9.**
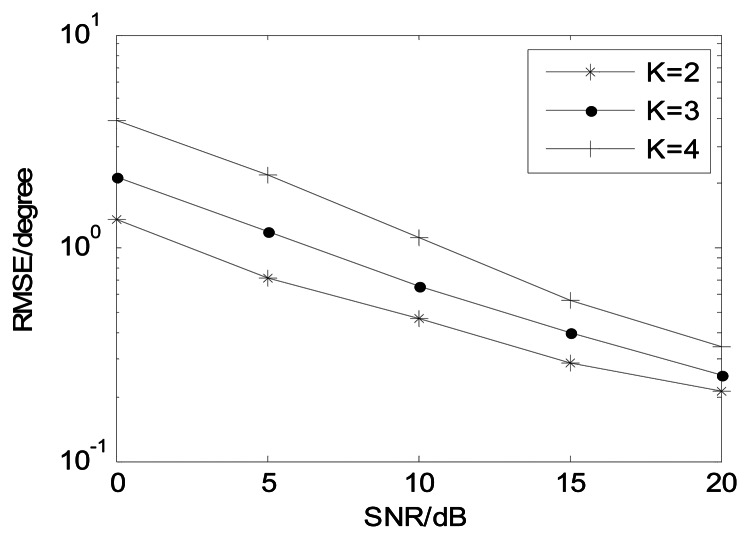
Angle estimation performance of our algorithm with different *K* (*M* = 12, and *J* = 100).

Figure 8 depicts the proposed algorithm estimation performance with different *J* (*M* = 12, and *K* = 3). It illustrates that the estimation performance becomes better in collaboration with *J* increasing and the proposed algorithm is effective in small snapshots. Figure 9 shows angle estimation performance of the proposed algorithm with different *K* (*M* = 12 and *J* = 100). It is indicated that the angle estimation performance of our algorithm is gradually improving as the number of sources is reduced.

**Figure 10.**
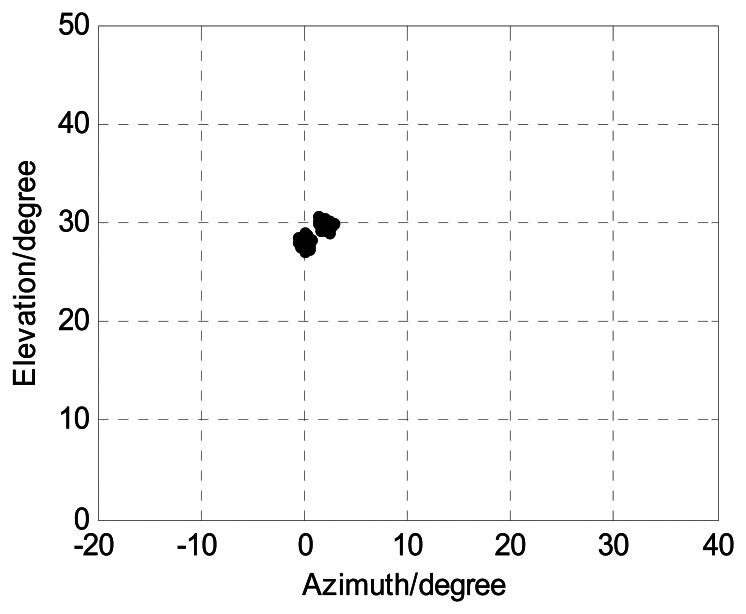
Angle estimation of our algorithm for closely spaced sources.

We assume two coherent closely spaced sources located at angles (*ϕ*_1_,*φ*_1_) = (0°,30°), (*ϕ*_2_,*φ*_2_) = (2°,28°). Figure 10 displays angle estimation of closely spaced sources exploiting the proposed algorithm with *M* = 12, *J* = 100, *K* = 2, and SNR = 15 dB. It implies that our angle algorithm has considerably good performance for angle estimation of closely spaced sources.

**Figure 11.**
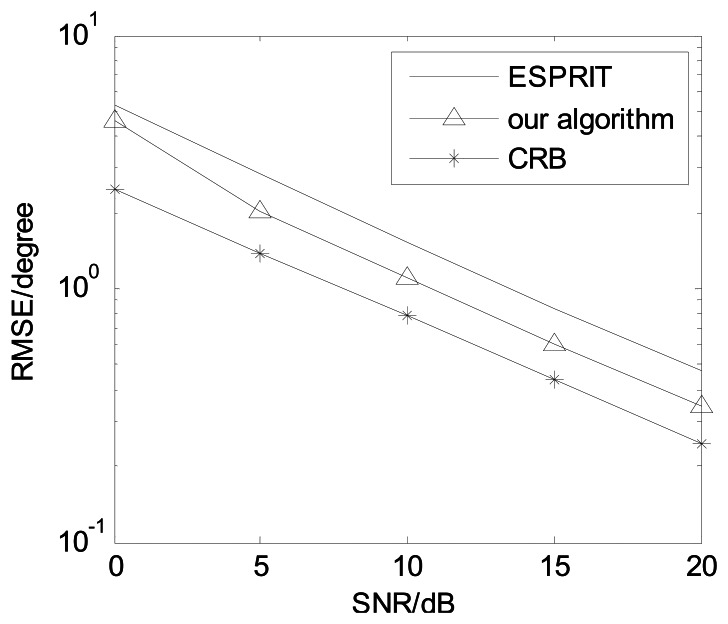
Angle estimation performance comparison for non-coherent sources.

For non-coherent sources, the proposed algorithm becomes the PARAFAC algorithm [14]. In non-coherent sources, we compared our algorithm against the ESPRIT algorithm [7] and CRB. Figure 11 presents angle estimation performance comparisons with *K* = 3, *M* = 8 and *L* = 150. It is indicated our algorithm has better DOA estimation performance than the ESPRIT algorithm [7].

## 5. Conclusions

In the present paper we have expanded the PARALIND model, originally applied to biology and chemistry, to acoustic vector-sensor array signal processing. The PARALIND model is used for coherent 2D- DOA estimation for arbitrarily spaced acoustic vector-sensor arrays subject to unknown locations. We have derived the PARALIND decomposition and its uniqueness in acoustic vector-sensor arrays, which is convenient for simulation, performance analysis and further study. Our algorithm obtains automatically paired 2D-DOA estimation and the correlated matrix of the sources. Compared with the FBSS-ESPRIT algorithm which only works for uniform arrays, our algorithm has much better angle estimation performance. Furthermore, our algorithm has considerably good performance for angle estimation of closely spaced sources.
